# Hysteroscopic polypectomy with vs. without levonorgestrel-releasing intrauterine system in women with endometrial polyps and dysmenorrhea

**DOI:** 10.1515/med-2026-1524

**Published:** 2026-10-12

**Authors:** Yongyan Ni, Yue Chen

**Affiliations:** Department of Obstetrics and Gynecology, Jiangsu Province Academy of Traditional Chinese Medicine, Nanjing, China

**Keywords:** endometrial polyps, hysteroscopic polypectomy, levonorgestrel-releasing intrauterine system, dysmenorrhea, menstrual blood loss, recurrence

## Abstract

**Objectives:**

To evaluate, among premenopausal women with endometrial polyps and documented dysmenorrhea, whether levonorgestrel-releasing intrauterine system (LNG-IUS) placement after hysteroscopic polypectomy (HP) was associated with better outcomes.

**Methods:**

This retrospective cohort study analyzed 276 premenopausal women with pathologically confirmed endometrial polyps who underwent HP between January 2022 and December 2024. Patients were classified into an HP-alone group (n=162) or an HP+LNG-IUS group (n=114).

**Results:**

At 12 months, the HP+LNG-IUS group had lower VAS scores than the HP group (aMD=−0.36; 95 % CI, −0.60 to −0.12; p=0.004). Clinically meaningful dysmenorrhea improvement occurred in 79.82 % in the HP+LNG-IUS group and 65.43 % in the HP group (aOR, 2.22; 95 % CI, 1.23–3.99; p=0.008). HP+LNG-IUS was also associated with a lower 12-month PBAC score and thinner endometrium. Exploratory analyses showed lower measured FSH, LH, estradiol, and progesterone levels. Polyp recurrence was observed in 2.63 % in the HP+LNG-IUS group and 9.26 % in the HP group.

**Conclusions:**

In this study, LNG-IUS placement after HP was associated with modestly lower pain scores, lower menstrual blood loss, thinner endometrium, and lower observed 12-month polyp recurrence. Residual confounding, sparse recurrence events, ultrasound-based recurrence ascertainment, and retrospective adverse-event capture limit causal and safety inferences. Prospective controlled studies are required.

## Introduction

Endometrial polyps are common benign endometrial lesions that may present with abnormal uterine bleeding, dysmenorrhea, infertility, or may be detected incidentally [1], [2], [3]. Symptomatic polyps are commonly treated by hysteroscopic polypectomy, which allows direct visualization, complete lesion removal, and histopathological confirmation [4], 5]. Although hysteroscopic treatment is effective, recurrent polyps and persistent symptoms remain clinically relevant. Previous adjunctive-treatment studies have focused mainly on recurrence. A meta-analysis of 19 randomized trials comparing LNG-IUS with oral progestins after hysteroscopic resection reported lower recurrence with LNG-IUS, and a retrospective cohort of 451 premenopausal patients reported first-year recurrence rates of 1.39 % with LNG-IUS and 6.19 % without LNG-IUS. However, pain, menstrual blood loss, and other patient-centered outcomes have been evaluated less consistently, leaving uncertainty about the incremental symptomatic value of immediate LNG-IUS placement after polypectomy [6], 7].

The levonorgestrel-releasing intrauterine system (LNG-IUS) delivers progestin locally to the uterine cavity and is widely used for heavy menstrual bleeding, dysmenorrhea-related conditions, and endometrial suppression [8], [9], [10], [11]. Its local progestogenic effect may reduce endometrial proliferation and menstrual blood loss, and this provides a biological rationale for its use after polypectomy as an adjunctive strategy for symptom control and recurrence prevention. However, the clinical value of combining hysteroscopic polypectomy with immediate LNG-IUS placement in patients with endometrial polyps remains incompletely defined, particularly in routine non-randomized practice.

Evidence regarding systemic ovarian effects of LNG-IUS is mixed. Barbosa et al. observed disturbed follicular development and lower preovulatory estradiol and LH peaks in some long-term users, whereas Xiao et al. found ovulation in 78.5 % of women after 6 years of use and concluded that the contraceptive effect was predominantly local. A randomized trial of 236 women treated for menorrhagia also demonstrated that hormone trajectories varied by treatment and follow-up time without establishing generalized hypothalamic–pituitary–ovarian axis suppression attributable to LNG-IUS. These inconsistent findings, together with the strong influence of menstrual-cycle timing on FSH, LH, estradiol, and progesterone measurements, justified exploratory hormone testing in the present cohort [12], [13], [14].

The primary objective was to evaluate the association between LNG-IUS placement after hysteroscopic polypectomy and 12-month dysmenorrhea severity in women with documented preoperative dysmenorrhea. Secondary objectives were to assess clinically meaningful pain improvement, menstrual blood loss, endometrial thickness, observed polyp recurrence, and recorded adverse events. Serum FSH, LH, estradiol, and progesterone were examined as exploratory outcomes because the systemic ovarian effects of LNG-IUS remain uncertain and cycle-phase standardization was incomplete.

## Materials and methods

### Study design and ethical approval

This retrospective cohort study was conducted at Jiangsu Province Academy of Traditional Chinese Medicine in China. The study protocol was reviewed and approved by the Institutional Review Board of the hospital ([DAS-2905-GM-04]). Written informed consent was waived because the study used anonymized data collected during routine medical care and no additional intervention was performed. All patient data were de-identified before analysis, and the study was conducted in accordance with the Declaration of Helsinki. The reporting was revised in accordance with STROBE principles for observational cohort studies (Table S1).

### Study population

Clinical data were collected from patients who underwent hysteroscopic endometrial polypectomy for pathologically confirmed endometrial polyps between January 2022 and December 2024. Inclusion criteria were: (1) adult premenopausal women; (2) diagnosis of endometrial polyps confirmed by preoperative transvaginal ultrasound and postoperative histopathological examination; (3) documented dysmenorrhea before treatment; (4) completion of 12 months of postoperative follow-up; and (5) availability of clinical records required for the study outcomes. The available institutional cohort was symptom-enriched and included only women with documented dysmenorrhea because repeated VAS measurements were required to evaluate a patient-centered outcome that had not been emphasized in recurrence-focused studies. Consequently, the study population does not represent all women undergoing polypectomy for endometrial polyps, particularly asymptomatic patients or those presenting only with infertility or abnormal bleeding.

Exclusion criteria were: (1) histopathological diagnosis of endometrial hyperplasia with atypia or endometrial malignancy; (2) other uterine pathologies, including submucous leiomyomas, adenomyosis, or intrauterine adhesions identified by preoperative hysteroscopy or imaging; (3) use of hormonal medications, including oral contraceptives, progestins, or gonadotropin-releasing hormone agonists, within 3 months before surgery; (4) contraindications to LNG-IUS placement, including uterine anomalies, active pelvic inflammatory disease, known hypersensitivity to levonorgestrel, or history of ectopic pregnancy; (5) pregnancy or lactation at enrollment; and (6) incomplete medical records or loss to follow-up. Known structural uterine causes of pelvic pain, including adenomyosis, submucosal leiomyomas, and intrauterine adhesions, were excluded using the available imaging and hysteroscopic records. Endometriosis status was not consistently documented in the retrospective dataset and therefore could not be used as a systematic exclusion criterion.

A total of 296 patients were screened for eligibility. Twenty patients were excluded: eight had incomplete follow-up data, six had coexisting adenomyosis, four had preoperative hormonal medication use, and two had histopathological endometrial hyperplasia. Consequently, 276 patients were included in the final analysis: 162 in the HP group and 114 in the HP+LNG-IUS group (Figure 1).

**Figure 1:**
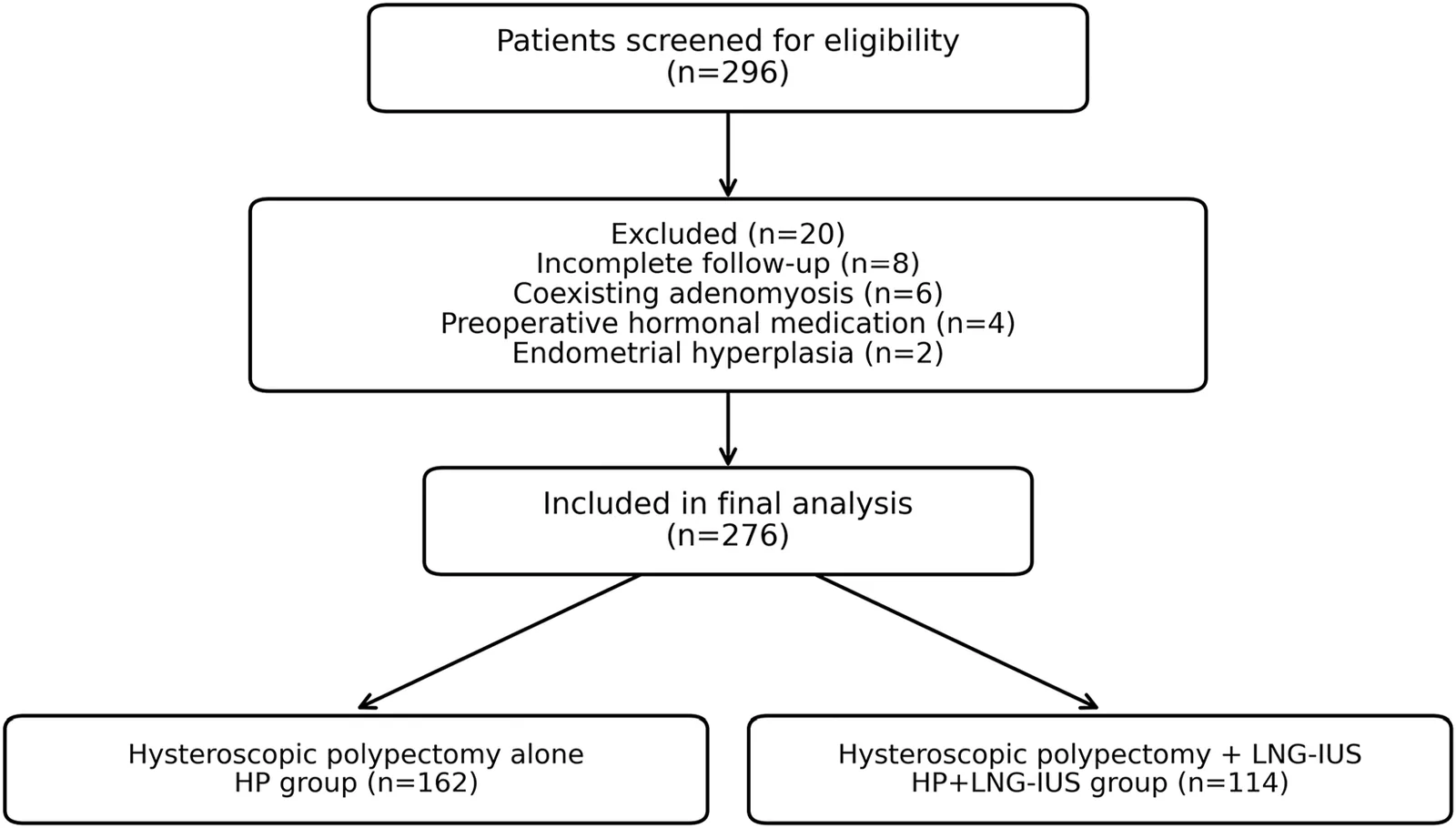
Patient flow diagram. HP, hysteroscopic polypectomy; LNG-IUS, levonorgestrel-releasing intrauterine system.

### Group allocation and interventions

All included patients underwent standard hysteroscopic endometrial polypectomy under general anesthesia. Patients were classified into two groups according to postoperative management: HP alone or HP combined with immediate LNG-IUS placement. Allocation was not randomized. It was based on patient preference after counseling about the benefits and risks of LNG-IUS and on routine clinical judgment. Clinical factors considered in routine practice may include baseline bleeding and pain severity, polyp burden, perceived recurrence risk, history of prior polypectomy, desire for long-acting reversible contraception, preference to avoid systemic hormonal treatment, and willingness to accept irregular bleeding or amenorrhea. Measured factors relevant to treatment selection – including baseline pain and bleeding scores, polyp burden, maximum polyp diameter, and previous polypectomy – were incorporated into adjusted analyses. However, contraceptive need, fertility plans, hemoglobin or anemia status, and the individual clinician’s treatment preference were not consistently recorded and could not be adjusted for. Therefore, this allocation process reflects real-world practice but creates potential selection bias and confounding by indication.

In the HP+LNG-IUS group, Mirena^®^ (Bayer HealthCare, Berlin, Germany), containing 52 mg levonorgestrel, was inserted immediately after polypectomy according to the manufacturer’s standard insertion protocol. Correct device position was confirmed intraoperatively in all 114 LNG-IUS recipients. Follow-up ultrasonography assessed device position when clinically indicated rather than through a systematic position-confirmation protocol; the proportion with documented follow-up position confirmation could not be retrieved from the medical records.

### Data collection and missingness

Clinical, laboratory, and ultrasonographic data were abstracted from the electronic medical record and routine follow-up documentation at baseline and at approximately 6 and 12 months. The analyzed cohort was a complete-case cohort by design: eight screened patients were excluded because 12-month follow-up was incomplete. Among the 276 included patients, no analysis fields were missing for VAS, PBAC, endometrial thickness, serum hormone measurements, recurrence, or recorded adverse events at the prespecified time points.

### Surgical procedure

Hysteroscopic endometrial polypectomy was performed during the early proliferative phase of the menstrual cycle, generally between cycle days 5 and 10, to optimize visualization. Procedures used a 4.5 mm rigid hysteroscope (Karl Storz, Tuttlingen, Germany) with a 5-Fr operating channel. Normal saline was used for uterine distension at 80–100 mmHg. Polyps were removed under direct visualization using a 5-Fr bipolar electrode loop (Versapoint; Gynecare, Somerville, NJ, USA), and the cavity was reinspected to confirm complete removal and exclude residual lesions. All specimens were sent for histopathological examination.

### Outcomes

The primary endpoint was dysmenorrhea severity at 12 months, measured using a 10-cm VAS ranging from 0 (no pain) to 10 (worst imaginable pain) [15]. Patients were instructed to report typical menstrual pain intensity during the preceding three menstrual cycles when menstrual cycles were present. Clinically meaningful dysmenorrhea improvement was recalculated directly from raw VAS values and defined as either a reduction of at least three points from baseline or a final 12-month VAS score of 3 or less.

Menstrual blood loss was assessed using the PBAC, originally described by Higham et al. [16]. Patients documented pad use, saturation, and clots for menstrual cycles at baseline and follow-up. Higher PBAC scores indicate greater menstrual blood loss. PBAC values were extracted as recorded in the records. The retrospective source data did not preserve cycle-level diaries or specify the scoring rule used for intervals in which amenorrhea developed; the recorded values were therefore not retrospectively recoded as zero. To evaluate the influence of this ambiguity, a sensitivity analysis excluded all patients who developed amenorrhea.

Endometrial thickness was measured by transvaginal ultrasound using a Voluson E8 system (GE Healthcare, Chicago, IL, USA) with a 5–9 MHz transvaginal probe. Measurements were obtained during the mid-luteal phase when cycles were present. For women with amenorrhea or irregular bleeding, measurements were performed at scheduled follow-up and interpreted with caution because cycle timing could not be standardized.

Peripheral venous blood samples were collected after overnight fasting. In menstruating patients, samples were planned for cycle days 3–5. In patients with amenorrhea, sampling was performed at the scheduled visit on a non-cycle-specific day. Serum FSH, LH, E2, and progesterone were measured by chemiluminescence immunoassay using an automated MAGLUMI 2000 Plus analyzer (Snibe Diagnostics, Shenzhen, China). Because amenorrhea and non-standardized sampling could influence measured hormone values, hormone results were considered secondary and exploratory, and a sensitivity analysis excluding amenorrheic patients was conducted. Moreover, because cycle-day confirmation was incomplete for some follow-up samples, these analyses estimate differences in measured hormone concentrations and cannot establish systemic hypothalamic–pituitary–ovarian axis suppression.

Endometrial polyp recurrence was assessed using transvaginal ultrasound at 6 and 12 months. Recurrence was defined as one or more newly detected endometrial polyps with a diameter of at least 5 mm after complete hysteroscopic polypectomy. Diagnostic hysteroscopy was performed when clinically indicated. Adverse events during 12 months were collected from clinical records and follow-up documentation, including irregular uterine bleeding, amenorrhea, pelvic pain, low back pain/discomfort, and LNG-IUS expulsion. Irregular uterine bleeding was defined as unscheduled bleeding or spotting for at least 3 days within a 30-day period during the first 6 months. Amenorrhea was defined as absence of menstrual bleeding for at least 90 consecutive days.

### Statistical analysis

All analyses were conducted using SPSS v.28. Continuous variables are presented as mean±standard deviation (SD), and categorical variables are presented as number and percentage. Normality was assessed using the Shapiro-Wilk test. Because several outcomes were non-normally distributed or bounded, crude continuous comparisons used Welch *t* tests and adjusted linear models used HC3 robust standard errors. Categorical variables were compared using Pearson chi-square tests when expected cell counts were adequate and Fisher exact tests when expected cell counts were less than five.

Longitudinal continuous outcomes measured at baseline, 6 months, and 12 months were analyzed using generalized estimating equations with an exchangeable working correlation structure and robust sandwich standard errors to account for within-patient correlation. Models included treatment group, time, group-by-time interaction, age, BMI, parity, hypertension, diabetes, history of cesarean section, duration of dysmenorrhea, multiple polyps, maximum polyp diameter, previous polypectomy, baseline VAS, baseline PBAC, and baseline endometrial thickness. Follow-up outcomes were also analyzed using baseline-adjusted ANCOVA with HC3 robust standard errors. For hormone outcomes, the baseline value of the respective hormone was additionally included when it was not already part of the covariate set. Sensitivity analyses excluded the four patients who developed amenorrhea for PBAC and hormone outcomes.

For binary outcomes, crude risk ratios or other appropriate effect measures and 95 % confidence intervals were reported. Clinically meaningful dysmenorrhea improvement was analyzed using multivariable logistic regression. Because only 18 recurrences occurred, recurrence was analyzed using Firth penalized logistic regression restricted to treatment group, history of previous polypectomy, and multiple polyps, with profile-likelihood confidence intervals. Irregular bleeding and other rare adverse events were not entered into multivariable models and were compared using Fisher exact tests. A propensity-score inverse probability of treatment weighting sensitivity analysis used the measured baseline covariates; after weighting, recurrence was analyzed using a treatment-only weighted logistic model to avoid overfitting. No correction for multiple comparisons was applied, and secondary and hormone outcomes were interpreted as exploratory. All tests were two-sided, with p<0.05 considered statistically significant. No *a priori* sample-size calculation was performed because all consecutive eligible records within the prespecified study period were included; precision is therefore presented using 95 % confidence intervals.

## Results

### Study population and baseline characteristics

Our study included 276 patients: 162 in the HP group and 114 in the HP+LNG-IUS group. Baseline demographic and clinical characteristics were generally comparable between groups. History of previous polypectomy was present in 12 patients (7.41 %) in the HP group and nine patients (7.89 %) in the HP+LNG-IUS group (Table 1). Among the 276 patients included in the complete-case analysis, outcome data were available for all analyzed variables at baseline, 6 months, and 12 months. Eight otherwise screened patients had been excluded because 12-month follow-up was incomplete.

**Table 1:** Baseline demographic and clinical characteristics.

Parameter	HP (n=162)	HP+LNG-IUS (n=114)	Effect estimate (95 % CI)	p-Value
Age, years	38.61±6.23	39.15±5.97	MD=0.54 (−0.92 to 2.00)	0.468
Body mass index, kg/m^2^	23.78±2.14	23.92±2.05	MD=0.14 (−0.36 to 0.65)	0.574
Parity (number of full-term pregnancies)	1.48±0.32	1.52±0.28	MD=0.03 (−0.04 to 0.10)	0.354
Duration of dysmenorrhea, months	31.25±14.38	32.41±15.02	MD=1.16 (−2.39 to 4.72)	0.519
Maximum polyp diameter, mm	16.52±5.46	17.04±5.82	MD=0.52 (−0.85 to 1.89)	0.453
Hypertension	23 (14.20 %)	18 (15.79 %)	RR=1.11 (0.63–1.94)	0.714
Diabetes mellitus	13 (8.02 %)	10 (8.77 %)	RR=1.09 (0.50–2.36)	0.825
History of cesarean section	44 (27.16 %)	33 (28.95 %)	RR=1.07 (0.72–1.55)	0.744
Multiple polyps	84 (51.85 %)	61 (53.51 %)	RR=1.03 (0.82–1.29)	0.786
History of previous polypectomy	12 (7.41 %)	9 (7.89 %)	RR=1.07 (0.47–2.39)	0.880

### Dysmenorrhea severity and menstrual outcomes

Baseline VAS scores were similar between groups. At 12 months, the HP+LNG-IUS group had a lower VAS score than the HP group (2.65±0.88 vs. 3.01±1.08; crude mean difference, −0.37; 95 % CI, −0.60 to −0.13; p=0.002). In the adjusted ANCOVA model, the 12-month mean difference was −0.36 (95 % CI, −0.60 to −0.12; p=0.004). The GEE model also showed lower follow-up VAS in the HP+LNG-IUS group, with an estimated 12-month difference of −0.39 (95 % CI, −0.64 to −0.13; p=0.003) and a group-by-time interaction p=0.044 (Table 2 and Figure 2).

**Table 2:** Crude and adjusted clinical continuous outcomes.

Outcome/time	HP	HP+LNG-IUS	Crude MD (95 % CI)	Crude p	Adjusted MD (95 % CI)	Adjusted p
VAS score, baseline	6.05±1.25	6.12±1.18	0.07 (−0.22 to 0.36)	0.628		
VAS score, 6 months	3.35±1.12	2.98±0.95	−0.37 (−0.61 to −0.12)	0.004	−0.37 (−0.62 to −0.12)	0.004
VAS score, 12 months	3.01±1.08	2.65±0.88	−0.37 (−0.60 to −0.13)	0.002	−0.36 (−0.60 to −0.12)	0.004
PBAC score, baseline	128.45±34.78	131.22±36.15	2.77 (−5.80 to 11.34)	0.526		
PBAC score, 6 months	82.34±22.56	51.28±18.44	−31.06 (−35.94 to −26.19)	<0.001	−30.66 (−35.75 to −25.57)	<0.001
PBAC score, 12 months	76.51±20.83	42.35±15.67	−34.16 (−38.49 to −29.84)	<0.001	−34.53 (−38.85 to −30.21)	<0.001
Endometrial thickness (mm), baseline	10.28±3.15	10.56±3.24	0.28 (−0.50 to 1.05)	0.481		
Endometrial thickness (mm), 6 months	7.36±2.18	5.32±1.56	−2.05 (−2.49 to −1.60)	<0.001	−2.04 (−2.50 to −1.58)	<0.001
Endometrial thickness (mm), 12 months	7.03±2.05	4.85±1.42	−2.17 (−2.58 to −1.76)	<0.001	−2.17 (−2.60 to −1.73)	<0.001

**Figure 2:**
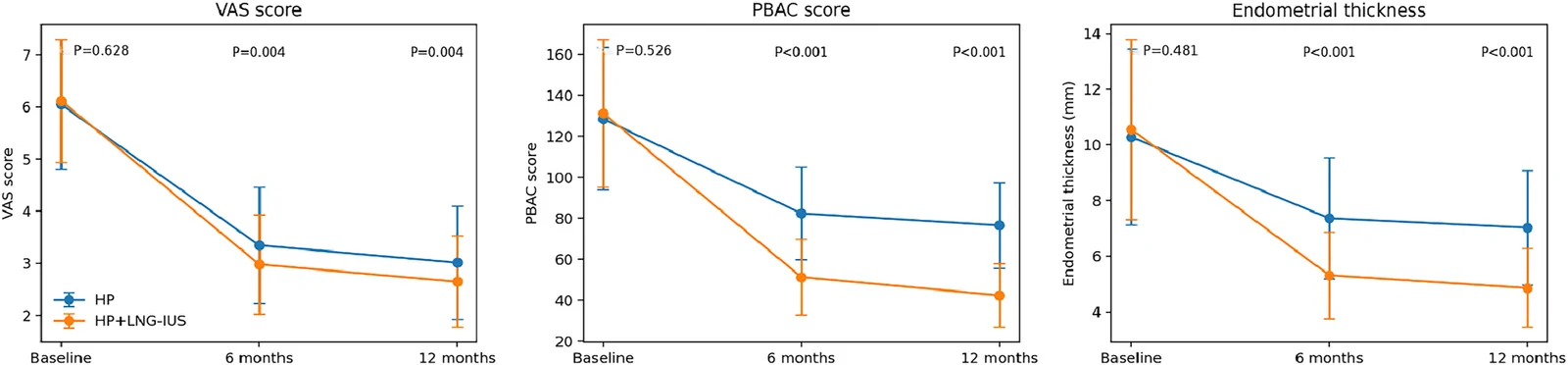
Longitudinal clinical outcomes. Points represent means and error bars represent standard deviations. p values compare groups at each time point; baseline p values are from Welch *t* tests and 6- and 12-month p values are from baseline- and covariate-adjusted ANCOVA models with HC3 robust standard errors. HP, hysteroscopic polypectomy; LNG-IUS, levonorgestrel-releasing intrauterine system; PBAC, pictorial blood loss assessment chart; VAS, visual analog scale.

Clinically meaningful dysmenorrhea improvement occurred in 91/114 patients (79.82 %) in the HP+LNG-IUS group and 106/162 patients (65.43 %) in the HP group (risk ratio, 1.22; 95 % CI, 1.06–1.41; p=0.009). The adjusted odds of clinically meaningful improvement were higher in the HP+LNG-IUS group (adjusted OR, 2.22; 95 % CI, 1.23–3.99; p=0.008) (Table 3).

**Table 3:** Endometrial polyp recurrence and adverse events during 12-month follow-up.

Outcome	HP	HP+LNG-IUS	Crude effect estimate (95 % CI)	p-Value	Adjusted OR (95 % CI); p-Value
Clinically meaningful dysmenorrhea improvement	106 (65.43 %)	91 (79.82 %)	RR=1.22 (1.06–1.41)	0.009	2.22 (1.23–3.99); p=0.008
Endometrial polyp recurrence	15 (9.26 %)	3 (2.63 %)	RR=0.28 (0.08–0.96)	0.028	Firth-adjusted OR 0.30 (0.08–0.88); p=0.028
Irregular uterine bleeding	3 (1.85 %)	8 (7.02 %)	Exact OR=3.79 (1.03–13.98)	0.056^a^	Not modeled because only 11 events occurred.
Amenorrhea	0 (0.00 %)	4 (3.51 %)	NA	0.028^a^	Not modeled because of zero events in the HP group.
Pelvic pain	2 (1.23 %)	0 (0.00 %)	NA	0.513^a^	Not modeled because of zero events in the HP+LNG-IUS group.
Low back pain/discomfort	1 (0.62 %)	2 (1.75 %)	2.84 (0.26–30.97)	0.571^a^	Not modeled because only 3 events occurred.
LNG-IUS expulsion	Not applicable	1 (0.88 %)			Descriptive outcome among LNG-IUS recipients.

^a^Fisher exact test. The recurrence adjusted analysis used Firth penalized logistic regression with treatment group, history of previous polypectomy, and multiple polyps. No adjusted model was fitted for irregular bleeding or other rare adverse events.

Menstrual blood loss was lower in the HP+LNG-IUS group at both follow-up visits. At 12 months, the adjusted mean difference in PBAC score was −34.53 (95 % CI, −38.85 to −30.21; p<0.001). Endometrial thickness was also lower in the HP+LNG-IUS group at both follow-up visits; the adjusted 12-month mean difference was −2.17 mm (95 % CI, −2.60 to −1.73; p<0.001) (Table 2, Figure 2). The repeated-measures GEE estimates for VAS, PBAC, and endometrial thickness are presented in Table 4. The PBAC findings were unchanged after excluding the four patients who developed amenorrhea. The adjusted between-group mean difference was −30.89 points at 6 months (95 % CI, −36.06 to −25.71; p<0.001) and −34.36 points at 12 months (95 % CI, −38.74 to −29.97; p<0.001) (Supplementary Table S2).

**Table 4:** Longitudinal GEE estimates for repeated continuous outcomes.

Outcome	6-month difference	12-month difference	Group × time p
VAS score	−0.39 (−0.65 to −0.13); p=0.003	−0.39 (−0.64 to −0.13); p=0.003	0.044
PBAC score	−31.97 (−37.68 to −26.26); p<0.001	−35.07 (−40.21 to −29.93); p<0.001	<0.001
Endometrial thickness, mm	−2.13 (−2.64 to −1.62); p<0.001	−2.26 (−2.73 to −1.79); p<0.001	<0.001
FSH, mIU/mL	−0.71 (−1.15 to −0.27); p=0.001	−0.68 (−1.08 to −0.27); p<0.001	0.110
LH, mIU/mL	−0.64 (−1.06 to −0.22); p=0.003	−0.61 (−1.00 to −0.22); p=0.002	0.083
Estradiol, pg/mL	−8.00 (−11.99 to −4.00); p<0.001	−8.52 (−12.27 to −4.76); p<0.001	0.013
Progesterone, ng/mL	−0.04 (−0.07 to −0.01); p=0.019	−0.05 (−0.08 to −0.02); p=0.002	0.035

### Measured serum sex hormone levels

Baseline hormone concentrations were similar between groups. At 6 and 12 months, the HP+LNG-IUS group had lower measured FSH, LH, estradiol, and progesterone concentrations in crude and adjusted analyses (Table 5 and Figure 3). At 12 months, the adjusted mean differences were −0.66 mIU/mL for FSH (95 % CI, −1.09 to −0.24; p=0.002), −0.62 mIU/mL for LH (95 % CI, −1.03 to −0.21; p=0.003), −8.77 pg/mL for estradiol (95 % CI, −12.75 to −4.80; p<0.001), and −0.05 ng/mL for progesterone (95 % CI, −0.08 to −0.02; p=0.003). Estimates remained directionally similar after excluding the four amenorrheic patients (Supplementary Table S2). These exploratory findings represent differences in measured concentrations and do not establish systemic ovarian-axis suppression because cycle-phase standardization was incomplete.

**Table 5:** Crude and adjusted measured serum sex hormone outcomes.

Outcome/time	HP	HP+LNG-IUS	Crude MD (95 % CI)	Crude p	Adjusted MD (95 % CI)	Adjusted p
FSH, mIU/mL, baseline	7.42±2.35	7.38±2.28	−0.04 (−0.59 to 0.52)	0.899		
FSH, mIU/mL, 6 months	6.15±1.92	5.43±1.76	−0.72 (−1.16 to −0.28)	0.001	−0.71 (−1.18 to −0.25)	0.003
FSH, mIU/mL, 12 months	5.76±1.85	5.08±1.64	−0.68 (−1.10 to −0.27)	0.001	−0.66 (−1.09 to −0.24)	0.002
LH, mIU/mL, baseline	5.68±2.42	5.75±2.35	0.07 (−0.50 to 0.64)	0.806		
LH mIU/mL, 6 months	4.82±1.85	4.15±1.68	−0.67 (−1.09 to −0.25)	0.002	−0.67 (−1.10 to −0.24)	0.002
LH, mIU/mL, 12 months	4.56±1.78	3.92±1.55	−0.64 (−1.04 to −0.25)	0.002	−0.62 (−1.03 to −0.21)	0.003
Estradiol, pg/mL, baseline	78.56±24.35	80.12±25.48	1.56 (−4.46 to 7.59)	0.610		
Estradiol, pg/mL, 6 months	60.38±18.56	52.24±15.42	−8.15 (−12.19 to −4.11)	<0.001	−7.75 (−12.01 to −3.49)	<0.001
Estradiol, pg/mL, 12 months	56.45±17.22	47.78±14.35	−8.67 (−12.42 to −4.91)	<0.001	−8.77 (−12.75 to −4.80)	<0.001
Progesterone, ng/mL, baseline	0.52±0.18	0.54±0.20	0.02 (−0.03 to 0.07)	0.405		
Progesterone, ng/mL, 6 months	0.38±0.15	0.34±0.14	−0.04 (−0.08 to −0.01)	0.016	−0.04 (−0.08 to −0.01)	0.024
Progesterone, ng/mL, 12 months	0.35±0.14	0.30±0.12	−0.05 (−0.08 to −0.02)	0.002	−0.05 (−0.08 to −0.02)	0.003

**Figure 3:**
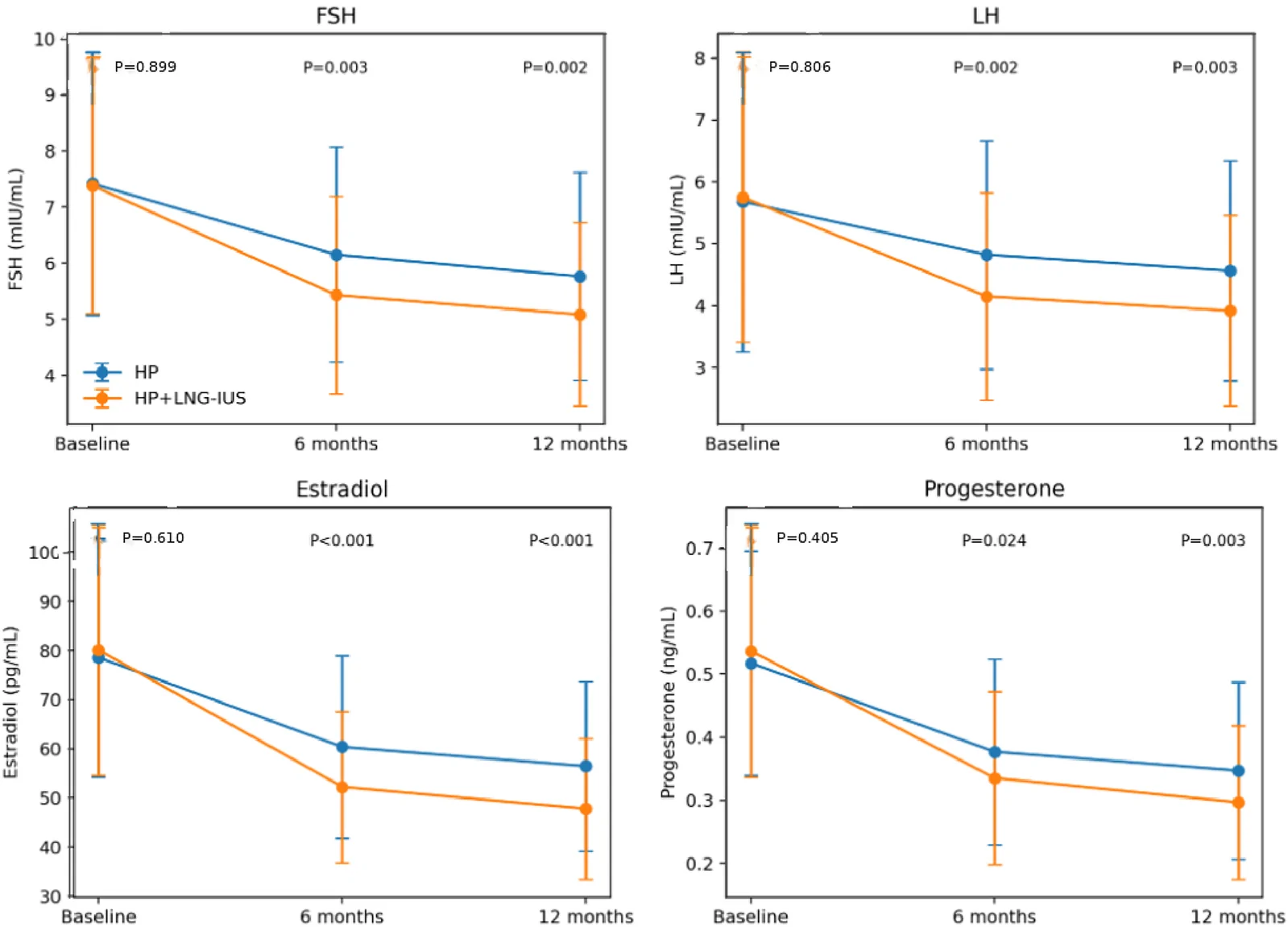
Longitudinal measured serum sex hormone concentrations. Points represent means and error bars represent standard deviations. p values compare groups at each time point; baseline p values are from Welch t tests and 6- and 12-month p values are from baseline- and covariate-adjusted ANCOVA models with HC3 robust standard errors. Hormone analyses are exploratory. E2, estradiol; FSH, follicle-stimulating hormone; HP, hysteroscopic polypectomy; LH, luteinizing hormone; LNG-IUS, levonorgestrel-releasing intrauterine system.

### Recurrence and adverse events

Polyp recurrence occurred in 15/162 patients (9.26 %) in the HP group and 3/114 patients (2.63 %) in the HP+LNG-IUS group (risk ratio, 0.28; 95 % CI, 0.08–0.96; risk difference, −6.63 %; 95 % CI, −11.97 % to −1.28 %; p=0.028). In the parsimonious Firth penalized logistic model adjusted for previous polypectomy and multiple polyps, HP+LNG-IUS remained associated with lower observed recurrence (adjusted OR, 0.30; 95 % profile-likelihood CI, 0.08–0.88; p=0.028) (Table 3).

Irregular uterine bleeding was more frequent in the HP+LNG-IUS group (8/114, 7.02 %) than in the HP group (3/162, 1.85 %), but the difference did not reach conventional statistical significance using Fisher exact testing (p=0.056). Because only 11 irregular-bleeding events occurred, no multivariable model was fitted. Amenorrhea occurred only in the HP+LNG-IUS group (4/114, 3.51 %; Fisher exact p=0.028). Pelvic pain, low back pain/discomfort, and expulsion were uncommon and are presented descriptively with Fisher exact comparisons where applicable (Table 3).

Propensity score inverse probability weighting achieved adequate balance for measured baseline covariates, with all standardized mean differences below 0.02 after weighting. Sensitivity analyses remained consistent with the main adjusted findings: the IPTW-adjusted 12-month mean differences were −0.37 for VAS, −34.42 for PBAC, and −2.16 mm for endometrial thickness. In the treatment-only weighted recurrence model, the odds ratio was 0.28 (95 % CI, 0.08–0.96; p=0.043). Propensity score balance before and after weighting is presented in Supplementary Table S3, and IPTW-adjusted sensitivity estimates are shown in Supplementary Table S4.

## Discussion

In this retrospective symptom-enriched cohort of 276 women with endometrial polyps and dysmenorrhea, LNG-IUS placement after hysteroscopic polypectomy was associated with modestly lower pain scores, a greater probability of clinically meaningful pain improvement, lower PBAC scores, thinner endometrium, and lower observed 12-month polyp recurrence. The patient-centered pain and bleeding outcomes extend a literature that has largely emphasized recurrence. The findings remained directionally consistent across robust regression, longitudinal, and propensity-weighted analyses. Nevertheless, non-random treatment allocation means these estimates describe associations rather than causal treatment effects.

The difference in 12-month VAS score was statistically significant but modest in magnitude. The adjusted mean difference was approximately 0.36–0.39 points, which may be below commonly used thresholds for a minimal clinically important difference in pain scores. Therefore, the clinical relevance of the mean VAS difference alone should not be overstated. In contrast, the proportion of patients meeting the defined clinically meaningful improvement criterion was higher in the HP+LNG-IUS group by approximately 14 percentage points, which may better reflect patient-level response.

The reduction in PBAC score and endometrial thickness is consistent with the known local progestogenic effect of LNG-IUS on endometrial proliferation and bleeding [8], [9], [10], [11, [17], [18], [19], [20], [21], [22]. These findings support the clinical plausibility of using LNG-IUS after polypectomy in selected patients with heavy menstrual bleeding or concern for recurrence. However, treatment selection may also have reflected contraceptive needs, fertility plans, baseline bleeding severity, anemia, recurrence-risk perception, patient tolerance of irregular bleeding or amenorrhea, and individual clinician preference. Several of these factors were not consistently recorded. Therefore, regression and propensity weighting address only measured confounding and cannot eliminate residual confounding by indication.

The 12-month recurrence rates in the present cohort – 2.63 % with HP+LNG-IUS and 9.26 % with HP alone – are directionally consistent with published evidence. A meta-analysis of 19 randomized trials by Sun et al. found a lower recurrence rate with LNG-IUS than with oral progestins after hysteroscopic resection [6]. In a retrospective cohort of 451 premenopausal patients, Wang et al. reported first-year recurrence rates of 1.39 % with LNG-IUS and 6.19 % without LNG-IUS, with cumulative recurrence rates during follow-up of up to three years of 3.47 and 15.96 %, respectively [7]. More recently, Peng et al., in a multicenter retrospective study of 1,160 patients, reported one-year recurrence rates of 5.17 % after HP+LNG-IUS and 13.55 % after HP alone [23]. These findings support the direction of the association observed in the present cohort. Nevertheless, direct numerical comparison is limited by differences in comparator treatment, patient selection, follow-up duration, and recurrence definitions. Furthermore, recurrence in the present study was primarily detected by ultrasonography, and only 18 recurrence events occurred. Therefore, the observed association requires confirmation in adequately powered prospective studies using standardized hysteroscopic or histopathological verification.

The hormone findings require particular caution. LNG-IUS acts predominantly through local endometrial exposure, and systemic suppression of the hypothalamic-pituitary-ovarian axis should not be inferred from these observational data alone [24], [25], [26], [27]. The lower measured FSH, LH, E2, and progesterone levels in the HP+LNG-IUS group may reflect cycle-timing differences, random-day sampling in amenorrheic patients, regression to the mean, or residual confounding. Sensitivity analyses excluding patients with amenorrhea produced similar results, but the absence of detailed cycle-day confirmation for every follow-up sample remains an important limitation. Therefore, these outcomes should be described as lower measured hormone levels rather than proven systemic hormonal suppression. Adverse events were generally uncommon, but the study was not powered to detect uncommon safety events. Irregular bleeding and amenorrhea were more common in the HP+LNG-IUS group, as expected for this device.

This study has several limitations. First, its retrospective, non-randomized design creates selection bias and confounding by indication. Although measured baseline clinical variables were adjusted for, contraceptive need, fertility plans, anemia or hemoglobin status, clinician preference, and other determinants of treatment choice were not consistently available. Second, the cohort included only women with documented dysmenorrhea, limiting generalizability to all patients with endometrial polyps; endometriosis status was also not systematically recorded. Third, the analysis was complete-case by design and eight patients with incomplete follow-up were excluded, so selection or attrition bias remains possible. Fourth, recurrence was uncommon, necessitating a parsimonious penalized model, and recurrence ascertainment relied mainly on ultrasound; patient-level hysteroscopic or pathological confirmation was unavailable. Fifth, adverse events were obtained from routine records rather than prospective surveillance, so uncommon harms may have been under-recorded. Sixth, follow-up device-position confirmation was not systematic, and unrecognized malposition could have influenced pain or bleeding outcomes. Finally, hormone analyses were exploratory because menstrual-cycle phase was not fully standardized. No *a priori* power calculation was performed, and the study may be underpowered for uncommon recurrence and safety outcomes.

## Conclusions

Among women with endometrial polyps and documented dysmenorrhea, LNG-IUS placement after hysteroscopic polypectomy was associated with modestly lower pain scores, reduced menstrual blood loss, thinner endometrium, and lower observed polyp recurrence over 12 months. Because the study was non-randomized and recurrence and adverse events were retrospectively ascertained, these findings should not be interpreted as causal or as establishing comparative safety. Prospective controlled studies with standardized recurrence confirmation are needed.

## Supplementary Material

Supplementary Material

## References

[1] Luo A, Shen W, Lv X, Yang S (2026). Endometrial polyps recurrence: risk factors, prevention, and management. Open Med.

[2] Reinikka S, Mehine M, Von Nandelstadh P, Ahvenainen T, Khamaiseh S, Nousiainen S (2025). Genomic landscape of endometrial polyps. Genome Med.

[3] Zhang L, Han Q, Bao MR, Wu Y (2025). Impact of endometrial polyps on pregnancy outcomes in patients with endometriosis and infertility: a systematic review and meta-analysis. Biomed Environ Sci.

[4] Li X, Zhao X, Wang B, Bai J, Qi Q, Li D (2025). Analysis of factors influencing endometrial polyp recurrence after hysteroscopic polypectomy. J Obstet Gynaecol.

[5] Xie A, Wu X, Su Y, Jia Y, Yang L, Liu Y (2025). Risk factors for endometrial polyps to transform into endometrial cancer: insights from a bibliometric analysis. J Health Popul Nutr.

[6] Sun Y, Chen X, Yuan X, Xu L, Jin Y, Ji T (2020). Levonorgestrel intrauterine system versus oral progestin for preventing the recurrence of endometrial polyps after hysteroscopic resection: a meta-analysis of 19 randomized controlled trials. Clin Exp Obstet Gynecol.

[7] Wang Y, Yang M, Huang X, Li X, Feng Y (2020). Prevention of benign endometrial polyp recurrence using a levonorgestrel-releasing intrauterine system in premenopausal patients: a retrospective cohort study. J Minim Invasive Gynecol.

[8] Fait T, Department of Gynecology and Obstetrics, 2nd Medical Faculty, Charles University and University Hospital Motol, Prague, Czech Republic (2024). Usage of the levonorgestrel releasing intrauterine system in perimenopause. Ceská Gynekol.

[9] Goldstuck ND (2023). The levonorgestrel-releasing intrauterine system 52 mg as a contraceptive versus a therapeutic: essential differences and perspectives. Eur J Contracept Reprod Health Care.

[10] Wei H, Pan N, Zhang W, Xiong G, Guo W, Dong Z (2023). Levonorgestrel-releasing intrauterine system-based therapies for early-stage endometrial cancer: a systematic review and meta-analysis. J Gynecol Oncol.

[11] Whitaker LH, Middleton LJ, Priest L, Odedra S, Cheed V, Nicholls EP (2023). Ulipristal acetate versus levonorgestrel-releasing intrauterine system for heavy menstrual bleeding: the UCON randomised controlled trial and mechanism of action study. Efficacy Mech Eval.

[12] Barbosa I, Bakos O, Olsson S-E, Odlind V, Johansson ED (1990). Ovarian function during use of a levonorgestrel-releasing IUD. Contraception.

[13] Xiao B, Zeng T, Wu S, Sun H, Xiao N (1995). Effect of levonorgestrel-releasing intrauterine device on hormonal profile and menstrual pattern after long-term use. Contraception.

[14] Halmesmaki K (2004). A randomized controlled trial of hysterectomy or levonorgestrel-releasing intrauterine system in the treatment of menorrhagia--effect on FSH levels and menopausal symptoms. Hum Reprod.

[15] Li L, Liu X, Herr K (2007). Postoperative pain intensity assessment: a comparison of four scales in Chinese adults. Pain Med.

[16] Higham JM, O’Brien PMS, Shaw RW (1990). Assessment of menstrual blood loss using a pictorial chart. BJOG.

[17] Atak Z, Rahımlı Ocakoğlu S, Ocakoğlu G (2023). Levonorgestrel-releasing intrauterine device to treat abnormal uterine bleeding; not one treatment option fits all. J Turk Ger Gynecol Assoc.

[18] Erkal N, Kaba M, Erdoğan K, Eryılmaz FEI, Sivri Z, Mayır YA (2026). Factors associated with hysterectomy after failure of levonorgestrel-releasing intrauterine system treatment in women with abnormal uterine bleeding. BMC Womens Health.

[19] Ganatra A, Shah F, Ganatra K (2024). Efficacy of levonorgestrel intrauterine system in the management of abnormal uterine bleeding: a retrospective analysis of a 100 women. J Obstet Gynaecol India.

[20] Kuzel D, Lisa Z, Fanta M, Zizka Z, Boudova B, Cindrova-Davies T (2022). Hysteroscopic management of endometrial polyps and submucous leiomyomas in women using a levonorgestrel-releasing intrauterine system. Minim Invasive Ther Allied Technol.

[21] Zhang Y, Wang X, Shan M, Lu Q, Tian T (2026). Efficacy of transcervical resection of polyp combined with levonorgestrel-releasing intrauterine system in the treatment of endometrial polyps: a randomized controlled trial. Minim Invasive Ther Allied Technol.

[22] Zhao R, Chen Y, Lu P, Hu Y, Jiang Q, Zhou Q (2026). Prediction of menstrual patterns and analysis of adverse effects for hysteroscopic endometrial polypectomy combined with LNG-IUS treatment: a single-center retrospective cohort study. Eur J Med Res.

[23] Peng C, Liu J, Ding H, Duan H, Chen L, Feng Z (2025). The effect of hysteroscopic endometrial polypectomy combined with LNG-IUS treatment on polyp recurrence: a multicenter retrospective study. Front Surg.

[24] Jensen JT, Reinecke I, Post TM, Lukkari-Lax E, Hofmann BM (2023). Extended use of levonorgestrel-releasing intrauterine system (LNG-IUS) 52 mg: a population pharmacokinetic approach to estimate in vivo levonorgestrel release rates and systemic exposure including comparison with two other LNG-IUSs. Contraception.

[25] Akhigbe RE, Afolabi OA, Adegbola CA, Akhigbe T, Oyedokun P (2024). Comparison of the effectiveness of levonorgestrel intrauterine system and dienogest in the management of adenomyosis: a systematic review and meta-analysis. Eur J Obstet Gynecol Reprod Biol.

[26] Da Costa Porto BT, Ribeiro PA, Kuteken F, Ohara F, Abdalla Ribeiro HS (2024). Levonorgestrel intrauterine system versus dienogest effect on quality of life of women with deep endometriosis: a randomized open-label clinical trial. Women Health.

[27] Guo W, Lin Y, Hu S, Shen Y (2023). Compare the efficacy of dienogest and the levonorgestrel intrauterine system in women with adenomyosis. Clin Ther.

