# Supplementary material for: Hysteroscopic polypectomy with vs. without levonorgestrel-releasing intrauterine system in women with endometrial polyps and dysmenorrhea
Source: Open Med (Wars). 2026 Oct 12;21(1):20261524. doi: 10.1515/med-2026-1524 (PMC13656975; doi:10.1515/med-2026-1524)
Supplement: Supplementary file 1 [file j_med-2026-1524_suppl_001.docx]

**Supplementary Tables**

Hysteroscopic polypectomy with versus without LNG-IUS in women with endometrial polyps

## Supplementary Table S1. STROBE checklist.

| **Section** | **Item** | **STROBE recommendation** | **Reported location** |
| --- | --- | --- | --- |
| Title and abstract | 1a | Indicate the study design using a common term in the title or abstract. | p.1: Title and Abstract |
| Title and abstract | 1b | Provide an informative, balanced summary of the study, methods, results, and conclusions. | p.1: Abstract |
| Introduction | 2 | Explain the scientific background and rationale for the investigation. | p.2: Introduction |
| Introduction | 3 | State specific objectives, including any prespecified hypotheses. | p.2: Introduction, final paragraph |
| Methods | 4 | Present key elements of the study design early in the paper. | p.2: Section 2.1 |
| Methods | 5 | Describe the setting, locations, relevant dates, recruitment/exposure periods, follow-up, and data collection period. | pp.2-3: Sections 2.1-2.2 |
| Methods | 6a | Give eligibility criteria and the sources/methods of participant selection and follow-up. | pp.2-3: Section 2.2; p.3: Figure 1 |
| Methods | 6b | For matched studies, give matching criteria and numbers of exposed/unexposed participants. | Not applicable |
| Methods | 7 | Define all outcomes, exposures, predictors, potential confounders, and diagnostic criteria. | pp.3-5: Sections 2.3, 2.5, 2.6 |
| Methods | 8 | For each variable of interest, give data sources and assessment/measurement methods; describe comparability across groups. | pp.3-5: Sections 2.3-2.5 |
| Methods | 9 | Describe efforts to address potential sources of bias. | pp.3,5; pp.9-10: Allocation, statistical methods, limitations |
| Methods | 10 | Explain how the study size was arrived at. | pp.2-3: Section 2.2 and Figure 1 |
| Methods | 11 | Explain how quantitative variables were handled in the analyses and whether groupings were chosen. | pp.4-5: Sections 2.5-2.6 |
| Methods | 12a | Describe all statistical methods, including methods used to control for confounding. | pp.4-5: Section 2.6 |
| Methods | 12b | Describe methods used to examine subgroups and interactions. | p.5: Section 2.6 |
| Methods | 12c | Explain how missing data were addressed. | pp.2-3 and Section 2.6 |
| Methods | 12d | For cohort studies, explain how loss to follow-up was addressed. | p.3: Section 2.2/Figure 1; p.10: Limitations |
| Methods | 12e | Describe any sensitivity analyses. | p.5: Section 2.6; pp.7,9: Results |
| Results | 13a | Report numbers of individuals at each stage: eligible, included, followed up, and analyzed. | p.3: Section 2.2/Figure 1; p.5: Section 3.1 |
| Results | 13b | Give reasons for non-participation or exclusion at each stage. | p.3: Section 2.2/Figure 1 |
| Results | 13c | Consider use of a flow diagram. | p.3: Figure 1 |
| Results | 14a | Give characteristics of study participants and information on exposures and potential confounders. | p.5: Table 1 |
| Results | 14b | Indicate the number of participants with missing data for each variable of interest. | Not applicable |
| Results | 14c | For cohort studies, summarize follow-up time. | pp.1,3-5 and Results tables |
| Results | 15 | Report numbers of outcome events or summary measures over time. | pp.5-9: Tables 2-5; Figures 2-3 |
| Results | 16a | Give unadjusted and adjusted estimates with precision, and specify confounders adjusted for. | pp.5-9: Tables 2-5; p.5: Section 2.6 |
| Results | 16b | Report category boundaries when continuous variables are categorized. | p.4: Section 2.5 |
| Results | 16c | When relevant, translate relative risk into absolute risk for a meaningful period. | p.9: Table 5 |
| Results | 17 | Report other analyses such as subgroup, interaction, and sensitivity analyses. | pp.7-9: Tables 4-5 and supplementary references |
| Discussion | 18 | Summarize key results with reference to study objectives. | pp.9-10: Discussion |
| Discussion | 19 | Discuss limitations, considering sources of potential bias or imprecision. | p.10: Discussion limitations paragraph |
| Discussion | 20 | Give a cautious overall interpretation considering objectives, limitations, multiplicity, similar evidence, and plausibility. | pp.9-10: Discussion and Conclusion |
| Discussion | 21 | Discuss generalisability/external validity of the study results. | pp.9-10: Discussion |
| Other information | 22 | Give the source of funding and the role of funders. | p. 10 |

## Supplementary Table S2. Sensitivity analysis excluding patients (n=4)who developed amenorrhea.

| **Outcome** | **Adjusted mean difference excluding amenorrhea (95% CI)** | **P-value** |
| --- | --- | --- |
| PBAC score, 6 months | -30.89 (-36.06 to -25.71) | <0.001 |
| PBAC score, 12 months | -34.36 (-38.74 to -29.97) | <0.001 |
| FSH (mIU/mL), 6 months | -0.70 (-1.17 to -0.24) | 0.003 |
| FSH (mIU/mL), 12 months | -0.67 (-1.11 to -0.24) | 0.002 |
| LH (mIU/mL), 6 months | -0.68 (-1.12 to -0.25) | 0.002 |
| LH (mIU/mL), 12 months | -0.64 (-1.05 to -0.23) | 0.002 |
| Estradiol (pg/mL), 6 months | -7.61 (-11.93 to -3.29) | <0.001 |
| Estradiol (pg/mL), 12 months | -8.64 (-12.64 to -4.63) | <0.001 |
| Progesterone (ng/mL), 6 months | -0.04 (-0.07 to -0.00) | 0.033 |
| Progesterone (ng/mL), 12 months | -0.05 (-0.08 to -0.02) | 0.004 |

*Note.* Adjusted mean differences compare the HP+LNG-IUS group with the HP group after excluding the four amenorrheic patients. Negative values indicate lower measured hormone levels in the HP+LNG-IUS group. CI, confidence interval; FSH, follicle-stimulating hormone; LH, luteinizing hormone; PBAC, pictorial blood loss assessment chart; VAS, visual analog scale.

## Supplementary Table S3. Propensity-score covariate balance before and after inverse probability of treatment weighting.

| **Covariate** | **SMD before weighting** | **SMD after IPTW** |
| --- | --- | --- |
| Age | 0.089 | 0.001 |
| BMI | 0.068 | 0.009 |
| Parity | 0.112 | 0.015 |
| Hypertension | 0.044 | 0.003 |
| Diabetes mellitus | 0.027 | -0.008 |
| History of cesarean section | 0.040 | -0.001 |
| Duration of dysmenorrhea | 0.079 | 0.009 |
| Multiple polyps | 0.033 | -0.001 |
| Maximum polyp diameter | 0.092 | -0.001 |
| Previous polypectomy | 0.018 | -0.007 |
| Baseline VAS score | 0.059 | -0.001 |
| Baseline PBAC score | 0.078 | 0.006 |
| Baseline endometrial thickness | 0.087 | 0.010 |

*Note.* Values are standardized mean differences (SMDs). Absolute SMDs closer to zero indicate better balance. All post-weighting absolute SMDs were below 0.02, indicating adequate balance across measured baseline covariates. IPTW, inverse probability of treatment weighting; PBAC, pictorial blood loss assessment chart; VAS, visual analog scale.

## Supplementary Table S4. IPTW-adjusted sensitivity analysis for key clinical outcomes.

| **Outcome** | **Effect measure** | **IPTW-adjusted effect (95% CI)** | **P-value** |
| --- | --- | --- | --- |
| VAS score, 12 months | Mean difference | -0.37 (-0.60 to -0.13) | 0.003 |
| PBAC score, 12 months | Mean difference | -34.42 (-38.74 to -30.10) | <0.001 |
| Endometrial thickness (mm), 12 months | Mean difference | -2.16 (-2.60 to -1.73) | <0.001 |
| Endometrial polyp recurrence | Odds ratio | 0.28 (0.08 to 0.96) | 0.043 |

*Note.* Mean differences compare the HP+LNG-IUS group with the HP group. Negative mean differences indicate lower 12-month values in the HP+LNG-IUS group. The recurrence estimate is presented as an odds ratio. CI, confidence interval; IPTW, inverse probability of treatment weighting; PBAC, pictorial blood loss assessment chart; VAS, visual analog scale.
